# Visceral Adipose Inflammation in Obesity Is Associated with Critical Alterations in Tregulatory Cell Numbers

**DOI:** 10.1371/journal.pone.0016376

**Published:** 2011-01-26

**Authors:** Jeffrey Deiuliis, Zubair Shah, Nilay Shah, Bradley Needleman, Dean Mikami, Vimal Narula, Kyle Perry, Jeffrey Hazey, Thomas Kampfrath, Madhukar Kollengode, Qinghua Sun, Abhay R. Satoskar, Carey Lumeng, Susan Moffatt-Bruce, Sanjay Rajagopalan

**Affiliations:** 1 Davis Heart and Lung Research Institute, The Ohio State University, Columbus, Ohio, United States of America; 2 Department of Surgery, The Ohio State University, Columbus, Ohio, United States of America; 3 Department of Pathology, The Ohio State University, Columbus, Ohio, United States of America; 4 Department of Pediatrics and Communicable Diseases, The University of Michigan, Ann Arbor, Michigan, United States of America; Dana-Farber Cancer Institute, United States of America

## Abstract

**Background:**

The development of insulin resistance (IR) in mouse models of obesity and type 2 diabetes mellitus (DM) is characterized by progressive accumulation of inflammatory macrophages and subpopulations of T cells in the visceral adipose. Regulatory T cells (Tregs) may play a critical role in modulating tissue inflammation via their interactions with both adaptive and innate immune mechanisms. We hypothesized that an imbalance in Tregs is a critical determinant of adipose inflammation and investigated the role of Tregs in IR/obesity through coordinated studies in mice and humans.

**Methods and Findings:**

*Foxp3*-green fluorescent protein (GFP) “knock-in” mice were randomized to a high-fat diet intervention for a duration of 12 weeks to induce DIO/IR. Morbidly obese humans without overt type 2 DM (n = 13) and lean controls (n = 7) were recruited prospectively for assessment of visceral adipose inflammation. DIO resulted in increased CD3^+^CD4^+^, and CD3^+^CD8^+^ cells in visceral adipose with a striking decrease in visceral adipose Tregs. Treg numbers in visceral adipose inversely correlated with CD11b^+^CD11c^+^ adipose tissue macrophages (ATMs). Splenic Treg numbers were increased with up-regulation of homing receptors CXCR3 and CCR7 and marker of activation CD44. *In-vitro* differentiation assays showed an inhibition of Treg differentiation in response to conditioned media from inflammatory macrophages. Human visceral adipose in morbid obesity was characterized by an increase in CD11c^+^ ATMs and a decrease in *foxp3* expression.

**Conclusions:**

Our experiments indicate that obesity in mice and humans results in adipose Treg depletion. These changes appear to occur via reduced local differentiation rather than impaired homing. Our findings implicate a role for Tregs as determinants of adipose inflammation.

## Introduction

Visceral adipose inflammation is believed to play an etiologic role in the development of insulin resistance (IR) in obesity and is typified by early, and often dramatic, increases in innate immune cells such as macrophages. Recently, multiple groups have demonstrated an increase in visceral adipose T lymphocyte subsets (ATL) in mouse and human obesity [1], [2], [3], [4]. The relationship between alterations in innate and adaptive immune cell numbers and function in the adipose and their contribution to the eventual development of obesity and related IR remains unclear. It has been suggested that sub-sets of infiltrating T cells may even be a primary event in the initiation of adipose tissue inflammation and development of IR [1], [2], [3]. In this regard, there is considerable controversy, over the precise subset of T cells that are important with some studies suggesting a role for CD8^+^ T [2] while others demonstrating a role for CD4^+^ T_H_1 cells [3]. Regulatory (Treg) cells may play a critical role in modulating levels of tissue inflammation via their interactions with several components of the immune system. A well defined role for these cells is in bridging interactions between responder/effector T cells, and antigen presenting cells (APCs) via cytokines such as IL-10 and TGFβ and direct cytostatic interactions [5], [6]. While Tregs have been demonstrated to play an important role in tolerance and auto-immunity in type I diabetes, several groups have recently postulated a role for Tregs in experimental models of type II DM and IR [3], [7]. However the origin of these cells, their functional determinants and whether or not a depletion of Tregs occurs *in-vivo* in humans is unclear. In this paper we demonstrate a role for Tregs *in-vivo* in humans and demonstrate in parallel studies in murine diet-induced obesity (DIO), that a reduction in adipose Tregs is associated with profound alterations in adipose tissue macrophages (ATMs) and effector T cell populations.

## Methods

### Ethical Approval

This study and its procedures were approved by the Committees on Use and Care of Animals and the Office of Responsible Research Practices, Human Institutional Review Board (IRB) of the Ohio State University under OSU protocol #2008H0177. Human informed consent was obtained in writing and a copy was inserted in the patients' medical records.

### Animals

Male Foxp3-GFP “knockin” mice (Foxp3gfp.KI) (N = 10 mice/diet group) were randomized to a standard chow (SCD) or a high fat diet (HFD – 60% energy from fat, Research Diets D12492) for 12 weeks. Before sacrifice, an intra-peritoneal glucose tolerance test was performed. At sacrifice, serum was collected for insulin ELISA (Crystal Chem Inc., IL USA). Homeostatic model assessment of insulin resistance (HOMA-IR) and quantitative insulin sensitivity check index (QUICKI) in mice were calculated as measures of beta cell function and insulin sensitivity, respectively [8]. Mice were kept on a 12/12 hr day/night schedule. Foxp3gfp.KI mice were a generous gift of Mohamed Oukka and Vijay Kuchroo at Brigham and Woman's Hospital Harvard Medical School. These mice express green fluorescent protein under control of a foxp3 promoter without altering foxp3 expression on a C57BL/6 background allowing *in-vivo* and *in-vitro* identification of Tregs (28).

### Human Participants

The study recruited and obtained visceral adipose samples from 20 surgical patients [7 lean (BMI<30), 7 obese non-treated (OB; BMI≥30), and 6 obese treated (OBTD) patients]. Samples were obtained from the greater omentum during endoscopic repair of hernias from lean subjects and during the performance of bariatric surgeries (OB/OBTD). The bariatric surgery performed consisted of laparoscopic banding in 10 subjects and gastric bypass in 7 subjects.

### Mouse and Human Adipose Digestion

After excision, adipose was well rinsed in PBS, minced, and digested with collagenase type II from *Clostridium histolyticum* (1 mg/ml) at 37°C, 140 rpm as detailed previously (22). The digesta was filtered through a 100 µm nylon cell strainer before centrifugation (300×g, 10 minutes). The resulting pellet is the stromal vascular fraction (SVF). Viable adipose tissue mononuclear cells were isolated from the SVF using Lympholyte M (Cedarlane Laboratories Ltd, Birlington, NC) in mice and Ficoll-Hypaque (GE Healthcare) in humans. Cells were washed and resuspended in flow buffer before counting and staining for flow cytometry. A portion was stored at −80°C in lysis buffer - Absolutely RNA Kit (Agilent Technologies, Stratagene).

### Splenic and mesenteric lymph node leukocyte isolation

Spleens and mesenteric lymph nodes [9] were isolated and physically disrupted with the end of a syringe plunger, suspended in complete DMEM growth media and then centrifuged at 300×g. The resulting pellet was re-suspended in 1× red blood cell lysis buffer (Biolegend), at room temperature with gentle shaking for 3 minutes followed by addition of 1× PBS and centrifugation. Cells were re-suspended in flow buffer (1× PBS, 5% FBS).

### Flow staining and cytometry

Cells were stained according to manufacturer's instructions. Briefly, approximately 1 µg of antibody was used per million cells followed by incubation at 4°C for 15 minutes. Cells were subsequently washed with flow buffer. GFP expressing cells were run immediately while other samples were resuspended in 1% neutral buffered formalin and analyzed by flow cytometry (BD FACS LSR II™ flow cytometer, Becton Dickinson, San Jose, CA). Data was analyzed using BD FACS Diva software (Becton Dickinson, San Jose,CA). All antibodies were purchased from Biolegend, Miltenyi Biotec, or BDbioscience.

### In-vitro Bone Marrow Derived Monocyte Culture and Activation

Primary bone marrow derived monocytes (BMDM) were cultured by flushing the femur and tibia with flow buffer, PBS with 5% FBS. The cells were resuspended in BMDM growth media (DMEM, 10% FBS plus L-cell conditioned media) in a CO_2_ incubator at 37°C for 5 days in a 150 mm suspension culture dish (Corning Incorporated, Corning, NY). At day 5 BMDMs cells were split into three groups: unstimulated control (UC), classically activated, and alternatively activated cells. Classically activated cells were primed with interferon gamma (R&D Systems IFNγ 485-MI) 200 U/ml for 10 hrs followed by stimulation by the addition of LPS (500 ng/ml) overnight. Alternatively activated cells were treated with interleukin 4 (IL4) 20 U/ml (R&D Systems, IL4 404-ML) overnight. The following day, the cells were washed with PBS, BMDM growth media was provided, incubated for 24 hrs, and collected for use in conditioned media experiments.

### CD4^+^ cell isolation and Treg differentiation

Splenic CD4^+^ cells from Foxp3gfp.KI mice were negatively selected using a Miltenyi Biotec system. Briefly, non-CD4^+^ splenic cells are labeled by a cocktail of biotin-conjugated monoclonal antibodies against mouse: CD8a, CD11b, CD45R, CD49b, and Ter-119. Cells are secondarily labeled with colloidal super-paramagnetic microbeads conjugated to a monoclonal anti-biotin antibody and run through a Miltenyi column. The magnetically labeled non-CD4^+^ T cells are retained in the column, while sterile CD4^+^ T cells run through. The cells are passed through another column to increase purity. CD4^−^ cells from the column flow-through were irradiated at 25 Gy. CD4^+^ T cells were grown in RPMI 1640 medium supplemented with 10 mM L-glutamine, 10 mM HEPES buffer, 1 mM sodium pyruvate, 1% penicillin/streptomycin, and 10% fetal bovine serum supplemented with 30% (v/v) of conditioned media from UC, CA, or AA groups (as described aboved) in the presence of irradiated CD4^−^ T cells and anti-mouse CD3ε antibody at 2 µg/ml. Cells were cultured for 72 hours, collected, washed, counted and stained with α-CD4 APC/Cy7 and analyzed by flow cytometry for GFP expression as a marker of Treg differentiation.

### Data Analysis

All data are expressed as mean ± SEM unless otherwise specified. Graphpad Prism software (Version 5) was used for statistical analysis using the student's t-test or one-way ANOVA and Boneferroni's post hoc test where appropriate. A P value of <0.05 was considered statistically significant.

## Results

### Diet-induced Obesity and Macrophage Inflammation in Mice

**Figure 1:**
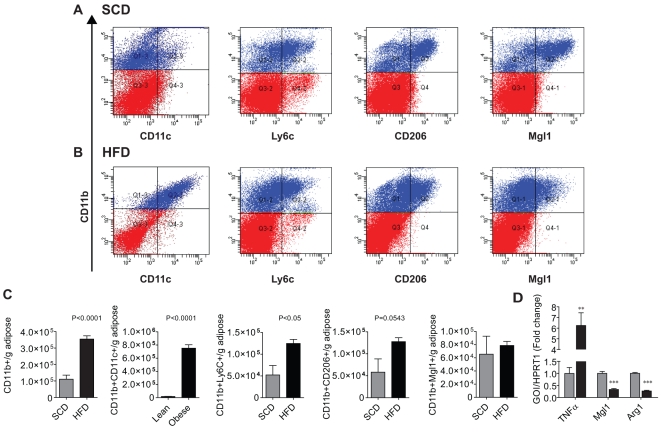
Diet-induced Obesity and Macrophage Inflammation in Mice. Representative flow cytometric dot plots showing double positive ATMs from Foxp3gfp.KI mouse epididymal adipose (N = 10/group) (A & B). The monocyte macrophage marker (CD11b^+^; y axis) is shown versus inflammatory (CD11c, Ly6c) and putative anti-inflammatory (CD206, Mgl1) markers (x axis). Blue indicates CD11b positivity within the live singlet gate (P1), red indicates P1 cells negative for CD11b. (C) There was a significant increase in inflammatory ATMs (CD11c^+^; Ly6C^+^) per gram of epididymal fat. Putative anti-inflammatory markers were not significantly affected; however, CD206 expressing cells increased (p = 0.0543). (D) There was a significant (**; p<0.005) increase in *TNFα* gene expression in the stromal vascular fraction with HFD and a significant (***; p<0.0005) decrease in *Mgl1* and *Arg1* expression (N = 6/group) as measured by real time PCR.

Foxp3gfp.KI mice developed insulin resistance after 12 weeks of HFD feeding as measured by intraperitoneal glucose tolerance test (IPGTT) (Figure S1), fasting insulin, HOMA-IR, and QUICKI (Table 1). Obese mice (46.2±0.9 g) weighed ≅70% more than lean controls (27.6±1.3 g). Flow cytometric analysis of visceral ATMs showed a ≅ four-fold increase in CD11b^+^ macrophages per gram (Figure 1A–C). CD11b^+^CD11c^+^ macrophages were dramatically increased with HFD. CD11b^+^Ly6C^+^ macrophages more than doubled in HFD diet mice, a pro-inflammatory subset that has been proposed to be recruited to visceral adipose tissue during obesity (Figure 1A–C) [10], [11], [12], [13], [14], [15]. We found an increase (p = 0.0543) in CD206^+^ expressing ATMs in this model though macrophage galactose N-acetyl-galactosamine specific lectin 1 (Mgl1) surface expression was not significantly different. To further characerize the macrophage activation state, real time gene expression analysis on the ATM/ATL fraction of the epididymal adipose was performed. *TNFα* increased ≅6-fold in DIO, indicating increased pro-inflammatory activation. *Mgl1* and *arginase 1* (*Arg1*) gene expression were signficantly (p<0.005) lower in the SVF of HFD mice as previously reported (Figure 1D) [16].

**Table 1:** Mouse metabolic parameters.

	SCD (n = 8)	HFD (n = 8)	P-value
Weight (g)	27.6±1.28	46.2±0.885	0.0001
Oral GTT (AUC)	23350±1276	44390±2505	0.0001
Fasting Glucose (mg/dl) [G]	81.5±7.83	141.33±7.74	0.0001
Fasting Glucose (mmol/L) [GO]	3.64±0.250	7.84±0.509	0.0001
Fasting Insulin (ng/ml)	0.476±0.220	2.48±0.378	0.0005
Fasting Insulin (mU/L) [IO]	14.5±5.18	61.5±9.38	0.0016
HOMA (GO*IO)/22.5	2.23±0.76	21.0±3.11	0.0003
QUICKI 1/[log (IO)+log(G)]	0.367±0.03	0.256±0.005	0.0053

### Lymphocytic Infiltration of Visceral Adipose in Obese Mice

**Figure 2:**
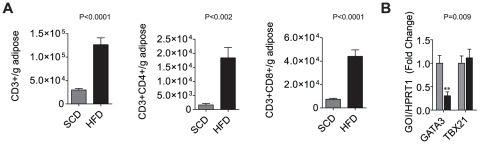
Lymphocytic Infiltration of Visceral Adipose in Obese Mice. (A) Flow cytometric staining for the lymphocytic markers CD3, CD4, and CD8 in Foxp3gfp.KI mice (N = 10/group) demonstrated a significant lymphocyte infiltration of the epididymal fat pad with obesity and insulin resistance. The CD8^+^ T cell population was >2-fold that of CD4^+^ per gram of adipose. (B) Gene expression of *GATA3* and *TBX21* in adipose-derived lymphoid cells from mouse epididymal fat following Lympholyte M isolation.

Total CD3^+^, CD3^+^CD4^+^, and CD3^+^CD8^+^ populations dramatically increased in the epididymal adipose with DIO (Figure 2A). It is important to note that CD8^+^ T cells numbers averaged ≅2-times that of CD4^+^ cells/gram in DIO. Gene expression of the T_H_2 marker *GATA3* in the SVF was ≅2-fold lower in the HFD (p<0.005) while expression of *Tbx21* remained comparable between the SCD and HFD groups (Figure 2B). There was no signficant difference in gene expression of *TGFβ1*, *IL6*, *IL4R*, and *IL12R* between groups (data not shown).

### Visceral Adipose Treg Deficiency in Obese Mice

**Figure 3:**
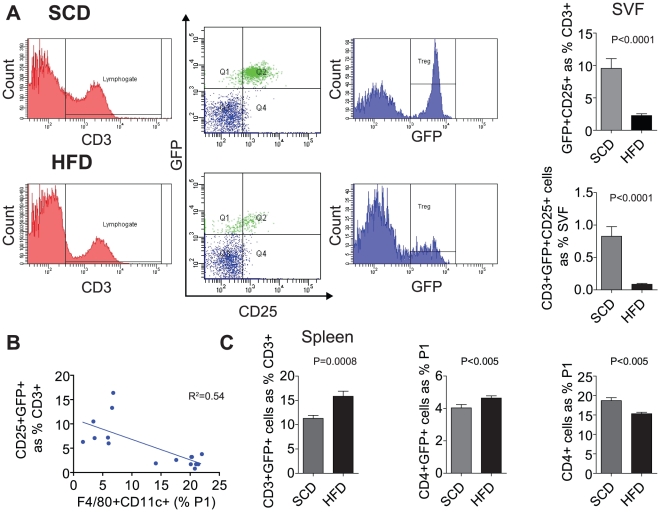
Obesity Mediates Local Treg Deficiency in the Visceral Adipose of Obese Mice. Visceral Treg numbers were examined by flow cytometry. (A) CD3^+^ T cells were identified followed by quantification of CD25^+^FoxP3^+^ Tregs as shown in Q2 of representative dot plots. HFD mice showed a 4-fold decrease in Tregs proportional to total CD3^+^ T cells. When normalized to percent of SVF, CD3^+^GFP^+^CD25^+^ decreased ≅ 10-fold. (B) There is an inverse relationship between the CD11c^+^ inflammatory ATMs and Tregs (R^2^ = 0.54). (C) HFD resulted in a significant increase in splenic CD3^+^GFP^+^ and CD4^+^GFP^+^ Tregs, with a concomitant (p<0.005) decrease in total splenic CD4^+^ T cells.

Figure 3A depicts Treg content of epididymal adipose from obese and lean animals. CD4^+^CD25^+^GFP^+^ Tregs decreased from 9.5% in SCD to 2.3% in the HFD group (p<0.0001, HFD vs. SCD). When normalized to epididymal adipose weight, Treg numbers were comparable between groups. The degree of Treg decrease was inversely correlated with the presence of CD11c^+^F4/80^+^ cells (Figure 3B, R ^2^ = 0.54, p<0.01). In order to determine if the depletion of Tregs in the adipose was a consequence of a systemic decrease in Tregs, we analyzed splenic content of Tregs. In contrast to the visceral adipose, there was a significant increase in CD3^+^GFP^+^ and CD4^+^GFP^+^ cells in the splenic response to HFD (Figure 3C, p<0.0008 & p<0.005 respectively vs. SCD) with a concominant decrease in total splenic CD4^+^ T cells (p<0.005).

### Splenic Treg Homing Receptor Expression in Obesity

**Figure 4:**
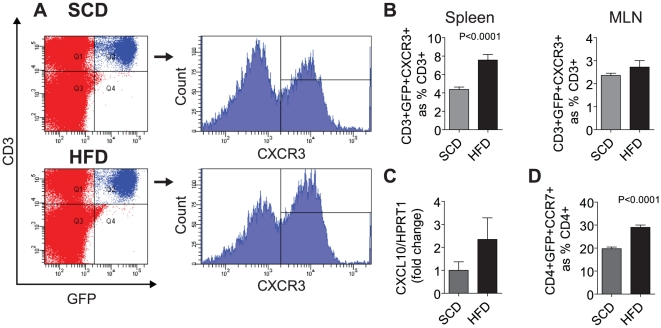
Splenic Treg Homing Receptor Expression in Obesity. The expression of receptors and cognate ligands involved in Treg homing were analyzed in the spleen and mesenteric lymph nodes (MLN, N = 10/group). (A) Representative dot-plot and histogram of Treg CXCR3 expression in SCD and HFD fed mice showing an increase in CXCR3 expressing Tregs in the spleen. (B) Analysis of splenic and mesenteric Tregs shows a non-significant increase in CXCR3 expression in splenic Tregs with no difference in MLN Tregs. (C) CXCL10 gene expression, the cognate ligand of CXCR3 receptor, was measure in the SVF of Foxp3gfp.KI mice. (D) Surface expression of the CCR7 receptor in splenic Tregs was significantly increased (p<0.0001) in the HFD group as measured by flow cytometry.

We hypothesized that the depletion of Tregs in fat may be related to obesity-induced alterations in chemokine receptor expression. Therefore, we analyzed surface expression of chemokine homing receptors CXCR3 and CCR7 on T cells in spleen and mesenteric lymph nodes (Figure 4A, B, D), two receptors previously described to regulate the trafficking of Tregs [17], [18], [19]. The percentage of splenic CXCR3^+^ Tregs nearly doubled in the HFD group compared to the SCD group (Figure 4A, B; p<0.0001). There was a non-significant increase in CXCR3 expression in mesentric lymph node-derived Tregs (Figure 4B). Gene expression of the cognate ligand of the CXCR3 receptor, CXCL10, in the adipose SVF was non-significantly increased in the HFD group (Figure 4C). CCR7 expression in splenic Tregs was markedly upregulated in response to HFD (Figure 4D; p<0.0001).

### Effects of HFD on T cell Activation

**Figure 5:**
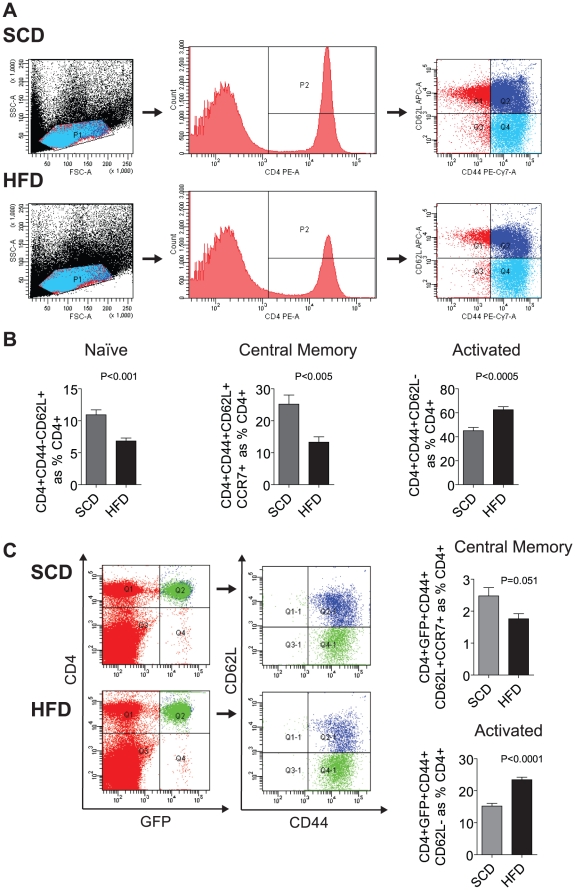
Diet-induced Obesity in Mice Activates Splenic T Helper and Tregs. The effect of DIO on CD4^+^ (A–D) and Tregs (E–G) sub-populations. (A) Representative dot-plot demonstrating isolation of CD4^+^ cells and analysis of expression of CD44, CCR7 (central memory cells) and CD62L (activation marker). (B) DIO resulted in a decrease in central memory and naïve T helper cells (p<0.005; p<0.001, respectively) and an increase in activated cells (p<0.0005) (C). Representative dot-plots of CD4^+^, GFP^+^ Tregs, showing CD44, CCR7 and CD62L expression. Splenic central memory Tregs (CD44^+^CD62L^+^CCR7^+^) showed a decrease (p = 0.051) while activated splenic Treg (CD44^+^CD62L^−^) were increased (p<0.0001).

To better understand the activation status of effector and regulatory T cells in the spleen, we analyzed surface expression of CD44 and CD62L (L-selectin) in Foxp3gfp.KI mice (Figure 5A). CD44 is upregulated on naïve T cells after T cell receptor activation and has been shown to be required for activated T cell extravasation into an inflammatory site [20], [21]. DIO resulted in a significant decrease in naïve CD4^+^ T cells (CD44^−^CD62L^+^; p<0.001) and central memory (CD44^+^CD62L^+^CCR7^+^; p<0.005) cells, but an increase in activated CD4^+^ cells (CD44^+^CD62L^−^; Figure 5B; p<0.0005). When the expression of CD44 and CD62L was examined in Tregs (CD4^+^ GFP^+^), we found that central splenic memory Tregs were non-significantly decreased (Figure 5C; p = 0.051) and activated splenic Tregs were increased (Figure 5C; p<0.0001). These results indicate that in response to DIO, there is an increase of activated T effector cells and Tregs in the spleen, suggesting that these cells are capable of mobilization to sites of inflammation or to appropriate chemokine gradients in peripheral tissue depots.

### Treg Differentiation is Inhibited by an Inflammatory Macrophage Milieu

Due to the paradoxical reduction of Tregs in visceral adipose in the face of increased Tregs in the spleen and activation of these cells in central reservoirs, we hypothesized that local activation state of adipose macrophages may critically impact T reg differentiation/maintenance. In order to test this hypothesis, we differentiated bone marrow derived macrophages from Foxp3gfp.KI mice and skewed their phenotype to a “pro-inflammatory” state using classic signals (LPS+IFNγ) or towards an “anti-inflammatory” activation state (IL-4). Culture media derived from these experiments were used in *in-vitro* Treg differentiation assays.

**Figure 6:**
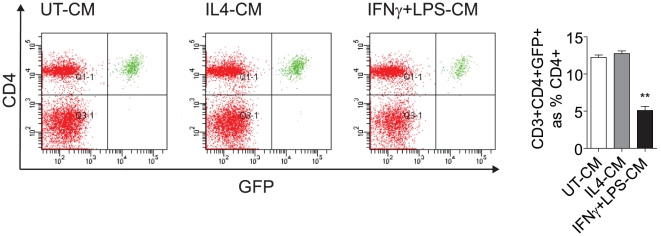
Treg Differentiation is Inhibited by an Inflammatory Macrophage Milieu. Splenic CD4^+^ cells from Foxp3gfp.KI were negatively selected in the presence of irradiated CD4^−^ T cells, anti-mouse CD3ε antibody, and conditioned media from untreated, IL4-, or IFNγ+LPS-differentiated bone marrow-derived macrophages. Cells were cultured for 72 hours, collected, stained, and analyzed by flow cytometry for CD4 and GFP expression as a marker of Treg differentiation. The IFNγ+LPS-CM group showed a significant (**, p<0.005) decrease in *in vitro* Treg differentiation when compared to the UT-CM group.

Figure 6 depicts *in-vitro* differentiation of CD4^+^CD3^+^GFP^−^ cells from the spleen of Foxp3gfp.KI mice in response to conditioned media derived from untreated, interleukin-4, or IFN-γ primed; LPS-stimulated macrophages. Approximately 12% of CD3^+^CD4^+^GFP^−^ cells grown *in-vitro* (Figure 6) in the presence of conditioned media from untreated bone marrow-derived macrophages differentiate to GFP^+^ Treg cells. In contrast, supplementation with conditioned media from IFNγ+LPS activated macrophages suppressed differentiation levels, with GFP^+^ cells reaching 5% (p<0.001 vs. unstimulated and alternatively activated groups). In the absence of bone marrow-derived macrophage conditioned media the differentiation rate was <4% (data not shown). Differentiation in the presence of conditioned media from IL-4-stimulated macrophages did not increase Treg differentiation beyond that of untreated media. This indicates that cytokines produced by IFNγ+LPS-stimulated macrophages prevent the differentiation of CD4^+^ T cell precursors to Tregs. It also suggests that mediators secreted from an alternately differentiated macrophage do not have a detrimental effect of T cell precursor cell differentiation to a Treg phenotype.

### Human Biomorphic Data

Table 2 summarizes the biometric and metabolic data in humans. Fasting glucose was highest in the obese-treated (OBTD) group (131±8.60 mg/dl, p<0.05 vs lean, obsese-nontreated), with the obese non-treated (OB) group (98.7±6.23 mg/dl) exhibiting fasting glucose in the normal range. Fasting insulin was signficantly higher in the OB and OBTD groups (p<0.05 vs lean). Hemoglobin A1c was non-significantly increased in the OBTD group. HOMA-IR values were significantly higher in the OB and OBTD groups (p<0.05 vs lean). C-reactive protein a sensitive marker of subclinical inflammation [22] was equally raised in both OB and OBTD groups, but did not reach signficance until both groups were combined (Obese – All, p<0.05).

**Table 2:** Human metabolic parameters.

	Lean (n = 5)	Ob (n = 7)	ObTd (n = 4)	Obese – All (n = 11)
Weight (lb)	169±11.1	264±14.9*	317±17.4*†,	290±19.2*
BMI	27.1±0.524	45.6±2.51*	49.4±1.97*	48.8±2.79*
Fasting Glucose (mg/dL)	90.4±9.08	98.7±6.23	131±8.60*,†	110±9.44*
Fasting Insulin (mU/L)	3.32±0.504	9.10±0.346*	13.7±0.478*	10.8±0.524*
Hem A1c (%)	5.56±0.114	5.61±0.148	7.20±1.00	6.29±0.649
QUICKI	0.425±0.0203	0.350±0.0163*	0.317±0.0150*	0.338±0.0165*
HOMA	0.760±0.287	2.21±0.450*	4.50±1.05*	3.04±0.824*
Total Cholesterol (mg/dL)	171±8.54	170±10.7	146±10.4	162±11.2
Triglycerides (mg/dL)	123±28.7	115±21.8	163±13.6	132±20.9
LDL (mg/dL)	102±9.87	113±10.4	85.8±4.29	103±10.1
HDL(mg/dL)	55.0±3.92	34.1±3.30	27.8±5.75	31.8±4.27
CRP (mg/dL)	2.36±0.734	9.26±2.07	8.97±4.07	9.15±2.74*

Fasting bloods were collected at time of surgery and sent to OSU clinical laboratories for analysis. Patient data were stratified to three groups (lean; OB, obese non-treated; OBTD, obese treated). Obese-ALL refers to the values and statistical analysis if patients were stratified by a BMI ≥30 alone, ignoring pharmaceutical treatments (*, p<0.05, group vs lean; †, p<0.05, OBTD vs OB).

### Human Obesity Results in Inflammatory Macrophage Infiltration of Visceral Adipose

**Figure 7:**
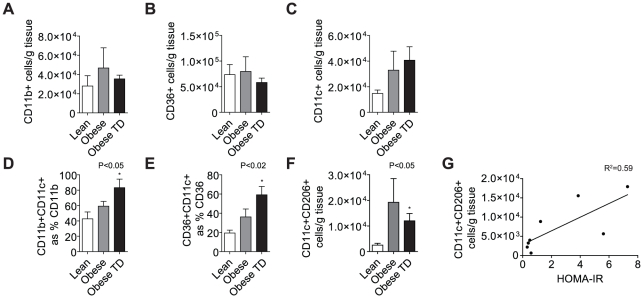
Human Obesity Results in Inflammatory Macrophage Infiltration of the Greater Omentum. ATMs were analyzed in the stromal vascular fraction of omental tissue from lean, obese and obese subjects who were being treated for insulin resistance (Obese TD). (A–B) depicts analysis of CD11b^+^ and CD36^+^ expressing ATMs/g of adipose tissue demonstrating no difference between groups. (C) CD11c^+^ cells per gram of adipose were non-significantly increase in both obese and obese TD groups. (D) CD11c^+^ cells expressed as percent of total CD11b^+^ and (E) as percent of CD36^+^ ATM demonstrating an increase in both obese and obese TD groups (p<0.05, vs lean). (F) CD11c^+^CD206^+^ ATMs were significantly increased (p<0.05) in the obese TD group, which was also characterized by the highest indices of insulin resistance (see table 2). (G) Linear regression plot of CD11c^+^CD206^+^ content in lean and obese TD individuals against HOMA-IR values (N = 4/group; R^2^ = 0.59).

We evaluated total, pro- and anti-inflammatory macrophage content of human greater omental fat, which is representative of visceral adipose tissue depots using several widely-used markers. Total macrophage content as measured by CD11b and the scavenger receptor (CD36) per gram of tissue was not increased in human obesity (Figure 7A, B). Interestingly, CD11c^+^ cells showed a trend toward an increase (Figure 7C). When the proportion of total ATMs was analyzed for CD11c^+^ expression, we found a significant increase in the CD11c^+^, pro-inflammatory macrophages (Figure 7D, E). Surface expression of CD11b^+^CD206^+^ cells, a putative surface marker of anti-inflammatory activation, on ATMs was not affected in morbid obesity, though we observed a non-significant (p = 0.0543) increase in the Foxp3gfp.KI mouse model [23]. Interestingly, CD11c^+^CD206^+^ cell content was significantly increased (Figure 7F; p<0.05) per grams of omentum in the OBTD group compared to lean controls; linear regression analysis of CD11c^+^CD206^+^ cells/g tissue with HOMA-IR values in these groups yielded a R^2^ value of 0.59 (N = 4/group).

### Activation Increases in Human Visceral Adipose CD4^+^ T cell with Obesity

**Figure 8:**
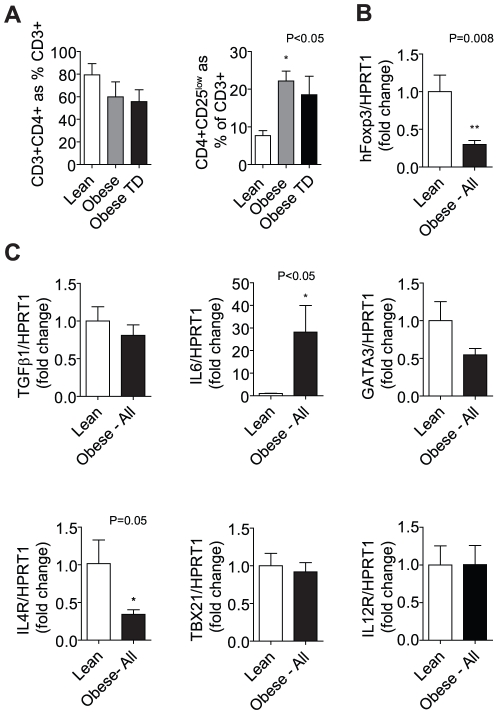
Human Obesity is Associated with Decreased *Foxp3* Expression. CD4^+^ T cells were non-significantly decreased in the greater omentum in obesity most probably due to an increase in CD8^+^ populations. Despite the decrease in total CD4^+^ cells there was a significant (p<0.05) increase in activated T effector cells (CD4^+^CD25^low^) as a percent of total CD3^+^ cells. (B) *Foxp3* gene expression was significantly (P = 0.008, N = 6/group) decreased in the obese state suggesting a decrease in Tregs. (C) *IL6* gene expression in the omentum was (p<0.05) significantly increased in the obese group. T_H_2 markers *GATA3* and *IL4R* were decreased, while T_H_1 markers were unchanged with obesity.

There was marked inter-sample variability amongst subjects when expressed per gram of tissue; CD4^+^ T cells, when expressed as a percentage of total CD3^+^, showed a non-significant decreased with human obesity (Figure 8A). There was no clear evidence of CD3^+^ cell infiltration in obesity when normalized to grams of adipose. CD25 is a traditional marker of T cell activation and is up-regulated in naïve, non-regulatory CD4^+^ T cells follow TCR engagement [24]. Activated lymphocytes as measured by CD3^+^CD4^+^CD25^lo^ cells however were dramatically increased from less than 10% in lean controls to greater than 20% in the OB (p<0.05). The obese TD group showed a non-significant increase similar to the OB group. CD3^+^CD4^+^CD25^hi^ cells were not readily evident in human samples; thus we examined *Foxp3* gene expression in human omental samples, which showed a significantly (p = 0.008) lower level of expression compared to lean controls (Figure 8B).

### Anti-Inflammatory T cell populations Decrease with Human Obesity

We then analyzed gene expression of T_H_1 and T_H_2 markers in human adipose to determine if there were differences in relative expression of pro- and anti- inflammatory T cell subpopulations markers. T_H_2 markers *IL4R* and *GATA3* were lower in the obese group compared to lean with no change in T_H_1 markers between groups (Figure 8C). This indicates that in addition to a loss of Treg populations there is a potential concomitant loss of other anti-inflammatory T cell subsets. GATA3, is a key transcription factor required for T_H_2 differentiation while the IL4 receptor is expressed on T_H_2 CD4^+^ T cells. In contrast *TBX21* gene expression (T-bet) a key regulator of T_H_1 differentiation was unchanged as was observed in the mouse. These changes suggest a skew towards a pro-inflammatory T cell phenotype within visceral adipose tissue by virtue of reduced anti-inflammatory (Treg and T_H_2) T cell populations. Together with a ≅30-fold higher IL-6 gene expression, these results are consistent with a pro-inflammatory phenotype of visceral adipose tissue.

## Discussion

In this paper we have demonstrated that visceral adipose depots are associated with reduction in Treg numbers. This deficiency in visceral adipose Tregs occurs in the context of increased splenic Tregs with up-regulation of key homing receptors on these cells. Treg differentiation was markedly impaired in response to culture in the presence of conditioned media from activated macrophages.

Visceral adipose inflammation in obesity is a major pathophysiologic risk factor for the development of whole body IR. Aberrant innate immune mechanisms have been postulated to be central to “metaflammation”, a sine que non of IR in obesity and Type II DM. Although Tregs have been investigated in animal models and human subjects with autoimmune conditions and type 1 diabetes, the role of Tregs in diet-induced experimental obesity has been only recently recognized [3], [7]. Moreover, the translatability of these findings to human obesity has not been evaluated. Our study provides new insights and extends prior observations that have suggested a role for T cell subsets in the pathogenesis of IR [2], [3], [7]. Treg depletion in visceral adipose tissue correlated with markers of innate immune activation and with the severity of IR at least in rodents. The murine model used in this study is a relevant model of diet-induced IR as evidenced by severe alterations in indices of glucose homeostasis and development of characteristic visceral adipose inflammation and infiltration by Ly6C^hi^ monocytes. Circulating Ly6C^hi^ monocytes are known to be recruited to sites of inflammation where they have been incriminated in genesis of pro-inflammatory tissue macrophages [15]. Our results are in keeping with recent data that have demonstrated an important role for Macrophage galactose-type C lectin 1 (Mgl1) as an important determinant of tissue infiltration by systemic monocytes [25], [26]. We found that CD11b^+^ cells expressing Mgl1 on their surfaces were not increased with DIO in our mouse model. Interestingly, Mgl1 gene expression in the SVF was significantly decreased in the HFD-mice. Although it is entirely likely that strain differences may have contributed to these findings, it is also possible that Mgl1 expressing ATMs in the visceral adipose at any given time may reflect a balance between infiltrating and resident macrophage populations.

The origin of adipose tissue Treg cells is currently not defined. It is clear that uncommitted, peripheral naïve CD4^+^ T lymphocytes can differentiate into Tregs based on mode of stimulation, antigen concentration and cytokine milieu [27]. The current model for Treg development suggests that “natural” Tregs generated in the thymus home to peripheral tissues to maintain self-tolerance [28], [29], [30]. There is also some data to support generation of Tregs (inducible Tregs) in the periphery during an active immune response [31], [32], though there are conflicting reports [33], [34]. In humans, it has been reported that CD25^+^ Tregs are derived from a pool of peripheral memory CD4^+^ T cells in addition to the thymus [35]. Upon observing a striking decrease in adipose Tregs in experimental obesity, we asked if this was due to inhibition of local Treg differentiation, alterations in homing, or a combination of both mechanisms. Peripheral Treg numbers in mice were inversely correlated with CD11c^+^ macrophages suggesting that there may be interactions between Tregs and macrophage/dendritic cells. Tregs directly interact with macrophage/dendritic cells in tissue niches where they inhibit T cell activation by blocking APC interactions with CD4^+^ effector T cells [3], [5], [6]. A loss of Tregs may potentiate inflammation through influences on macrophage activation state, which Tregs may influence via both cytokine-dependent (IL4, IL10, IL13) and independent pathways [36].

A reduction in Treg numbers or function may lead to pro-inflammatory skew in visceral adipose and may potentiate both innate and adaptive immune inflammation. Thus a decrease in Tregs may directly result in an increase in “activated” effector T cell populations and is supported by our studies in mice and humans that show higher activated effector T cell populations. In keeping with these data, Nishimura and colleagues demonstrate an improvement in IR genetic depletion of CD8^+^ cells while adoptive transfer of CD8^+^ T cells to CD8-deficient mice increased adipose inflammation [2]. Winer and colleagues recently reported a role for adipose infiltration by pro-inflammatory T_H_1 cells in the development of IR [3]. They reported improved long-term glucose and insulin homeostasis by CD3 antibody depletion in obese B6 mice with concomitant restoration of epididymal Tregs; treatment was less efficacious in an ob/ob model. However, adoptive transfer studies using CD4^+^Foxp3^−^ T cells to HFD-fed Rag1-null mice showed protection from weight gain and improvement in markers of glucose metabolism which tempered the hypothesized role for Tregs. Transfer of IL-10 null CD4^+^ T cells showed similar protection, indicating that Treg-mediated IL-10 production was not necessary in mediating beneficial effects. In contrast, a newly identified group of TGFβ-dependent regulatory T cells which express surface latency-associated peptide (LAP) have been shown by Ilan et al to pivotally modulate visceral adipose inflammation [7]. Induction of CD4^+^LAP^+^ Tregs by oral CD3 antibody as well as adoptive transfer of CD4^+^LAP^+^ Tregs resulted in amelioration of markers of insulin resistance in ob/ob mice. It is important to note that Winer also showed that adoptive transfer of CD4^+^ STAT6-null T cells to HFD-fed Rag1-null mice did not result in significant improvement in glucose metabolism as did WT CD4^+^ transferred cells. This was attributed to a reduced number of CD4^+^GATA3^+^ T_H_2 T cells generated from the STAT6-null adoptive transfer. Our data show that murine obesity lead to splenic CD4^+^ T cell activation.

CD44 expression is required for the development of memory T cells [37] and is increased upon T_H_1 cell activation and clonal expansion. Importantly, CD44 expression also mediates T cell migration and extravasation into tissues by acting as an adhesion molecule recognizing extracellular matrix molecules such as hyaluronin and protein ligands such as osteopontin that are produced during inflammation by T_H_1 cells [38]. At least 20 isoforms of CD44 have been described at the mRNA level [39]. A minimum of six CD44 protein products have been identified [40] though a standard 85–95-kDa form (CD44s) is the primary form expressed on lymphocytes [41], [42]. During T cell stimulation, expression of CD44 variants (v3, v6, v9) may be necessary for functional changes post-stimulation [43], [44]. It should be noted that the antibody we utilized has been reported to be specific to all CD44 isoforms. The role of CD44 splice variants in *in-vivo* T cell subpopulations is mostly likely of great immunological relevance. Thus, understanding *in-vivo* CD44 splice variant expression on T cell subsets during inflammatory processes such as obesity-mediated insulin resistance is an important area for further study.

Decreased *GATA3* expression in the SVF of obese mice and humans indicates a decrease in T_H_2 cells with the development of insulin resistance. The decrease in *IL4R* expression in obese humans also supports this hypothesis. Our data taken in the context of these reports indicates that there is a population shift from anti-inflammatory T cell subsets (Tregs, T_H_2 cells) to inflammatory T effector cell populations (T_H_1 cells) in the visceral adipose in obesity leading to the development of IR and Type II DM.

Conditioned media from IFNγ+LPS-activated macrophages reduced spontaneous Treg differentiation in a murine CD4^+^CD25^−^ T cell proliferation assay, while media from IL4-activated BMDMs insignificantly increased differentiation beyond that of untreated control media. This suggests that cytokines produced by infiltrating macrophages may play a role in suppressing local adipose Treg differentiation. To address the role of homing of peripheral Tregs to the adipose we examined splenic CXCR3 expression, a critical receptor required for T cell homing and found an increase (rather than decrease) in expression of this receptor in central lymphoid tissues. The lymphoid homing receptor, CCR7, was also increased in splenic Tregs in obesity. Taken together with the results suggesting a lack of effect of obesity in reducing central Treg numbers and homing receptors, we hypothesize that reduced Treg numbers in obesity may represent a functional consequence of macrophage derived inflammatory signals.

The studies in humans demonstrated a relatively more modest effect on inflammation based on absolute numbers of macrophages, though proportional increases in inflammatory macrophages were evident. We observed high within-group variability with respect to CD3^+^ cells/g and CD4^+^ cells/g that did not correlate with BMI. However, “activated” lymphocytes were consistently and statistically higher in the obese patients when compared to the lean human controls. There was a skew away from a T_H_2 phenotype towards a T_H_1 phenotype as evidenced by a marked decrease in *GATA3* and *IL4R* with constant levels of *TBX21* expression as described previously [45]. The expression of foxp3 in human omentum was diminished in keeping with results in our murine experiments. However in our study CD11c^+^CD206^+^ cells in humans were also increased, similar to the results reported in the mouse model. CD11c^+^CD206^+^ cells have recently been shown to play an important facilitatory role in adipose inflammation and insulin resistance but are unique as they also demonstrate features of alternatively activated macrophages such as high levels of IL10, high mitochondrial copy number, and scavenger receptor expression [23]. Thus, the binary M1/M2 paradigm in mouse ATMs may not be entirely applicable to humans. In keeping with these findings, the scavenger receptor CD36 was markedly increased in human obesity.

Temporal analysis of Tregs levels in the context of visceral immune and adipcyte phenotypes during DIO may have added valuable insights. The scope of the study was additionally limited as we did not perform interventions to modulate Tregs in visceral adipose tissue. Nevertheless, the use of human visceral adipose tissue from a patient population with morbid obesity (BMI>35) and an effective murine model to study Tregs in DIO allowed us to add clear data concerning the status of T cell and macrophage inflammation in the adipose. Further study is needed to characterize the direct and indirect interactions of dendritic, macrophage, and T cell subpopulations and the roles these interactions play in the etiology of DIO and IR. Better understanding may facilitate the development of a targeted pharmaceutical intervention that disrupts or enhances key interactions which modulate adipose inflammation resulting in improved systemic insulin resistance and metabolic syndrome.

## Supporting Information

Figure S1 Intraperitoneal glucose tolerance test (IPGTT) of Foxp3gfp.KI mice after 12 weeks on a HFD (60% energy from fat). HFD mice had higher fasting glucose and show impaired glucose plasma glucose clearance after IP dextrose bolus at 2 mg/g meeting the criteria for insulin resistance. HFD values reached statistical significance (p<0.0001) at all collection points when compared to SCD mice at corresponding intervals.
(EPS)
